# Transcecal endoscopic appendectomy and endoscopic submucosal dissection with hand-suturing–assisted traction and closure technique

**DOI:** 10.1016/j.vgie.2025.01.005

**Published:** 2025-01-17

**Authors:** Fatih Aslan, Serhat Ozer, Burcu Saka, Bahadir Hakan Oguz

**Affiliations:** 1 Koc University Hospital, Gastroenterology and Advanced Endoscopy, Istanbul, Turkey; 2 Koc University Hospital, Pathology Department, Istanbul, Turkey; 3 Koc University Hospital, Anesthesiology and Reanimation, Istanbul, Turkey

## Introduction

The treatment of cecal polyps involving appendiceal orifice and/or its lumen is technically challenging, given difficulties in clearly defining the boundaries.^1^ There is no consensus regarding the best treatment approach for these polyps. Surgical resection can provide a curative option; however, it carries a certain risk of morbidity. In recent years, several minimally invasive endoscopic treatment techniques have emerged and yielded successful outcomes in the management of appendix polyps. These include traditional polypectomy methods, EMR, endoscopic submucosal dissection (ESD), endoscopic full-thickness resection, and transcecal endoscopic appendectomy (TEA).^2^^,^^3^ Herein, we present a case of a polyp involving the lumen of the appendix.

## Case

**Figure 1:**
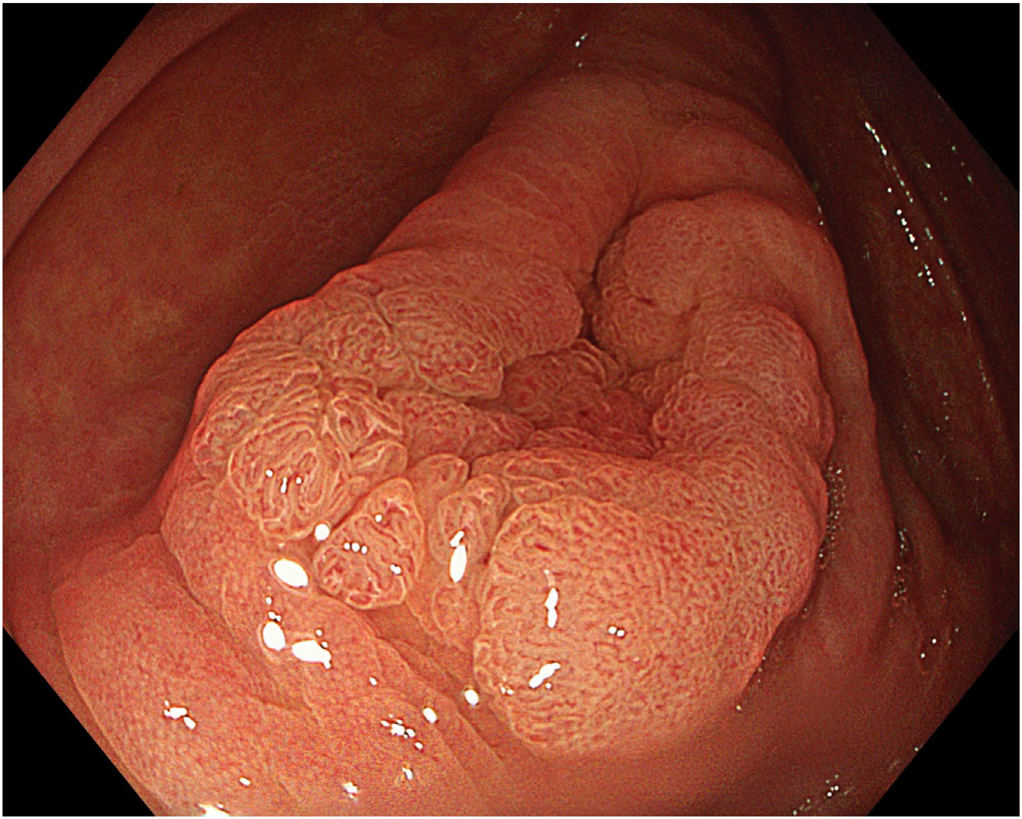
Endoscopic view of a cecal laterally spreading tumor extending into the appendix.

A 63-year-old male patient underwent colonoscopy (Olympus CF-EZ1500DL, Tokyo, Japan) upon altered bowel habits, and a granular laterally spreading lesion involving the cecum and appendix was detected. Lesion border within the appendix was not clearly defined. Lesion surface and vascular pattern were observed to be regular in texture and color enhancement imaging, narrow-band imaging, and extended depth-of-field modalities (Fig. 1). Abdominal tomography revealed neither distant metastasis nor lymph node involvement. With the patient under general anesthesia, the procedure involved hand-suturing traction,^4^ ESD, TEA, and closure, respectively. Prophylactic parenteral antibiotics (cefazolin sodium, 2 g/day, and metronidazole, 1000 mg/day) were administered and continued until discharge.

**Figure 2:**
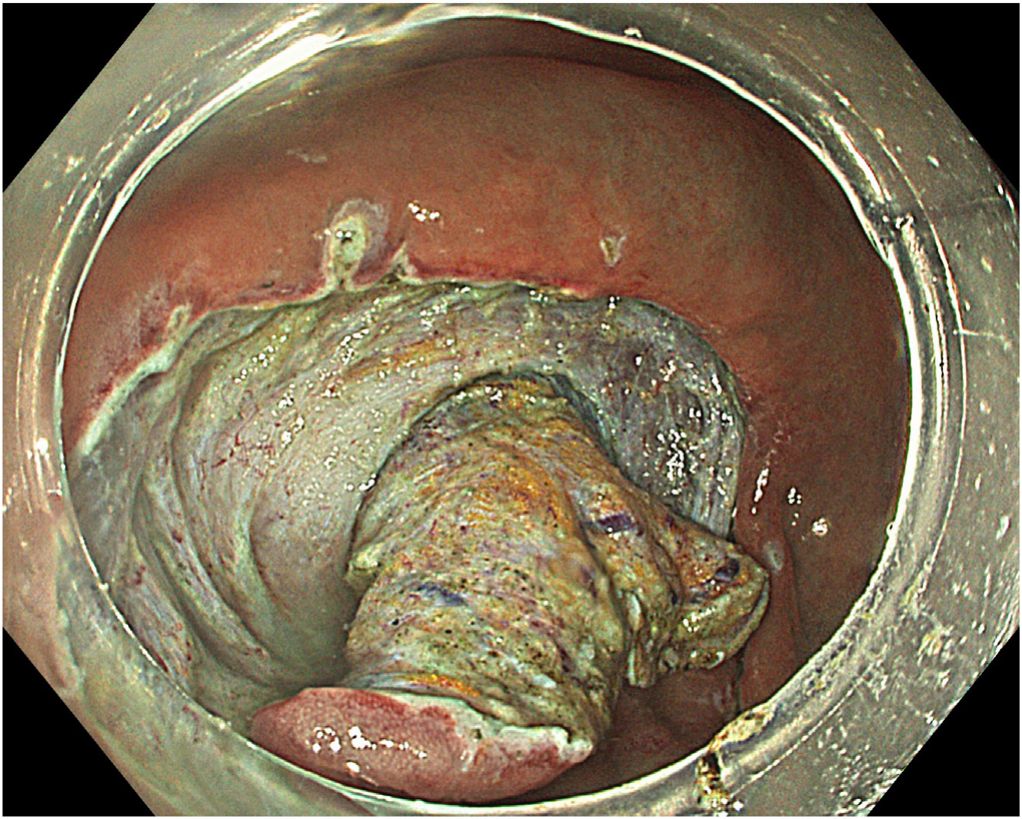
View of the lesion dissected up to the appendiceal orifice.

**Figure 3:**
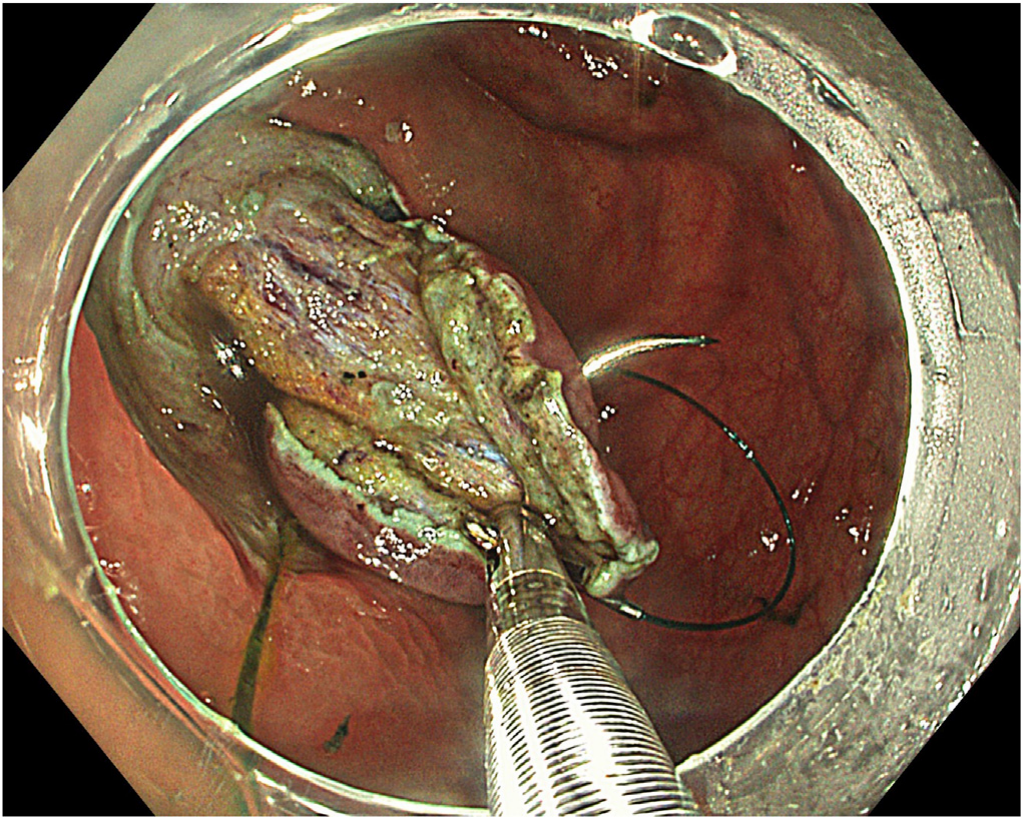
Application of barbed suture for traction.

**Figure 4:**
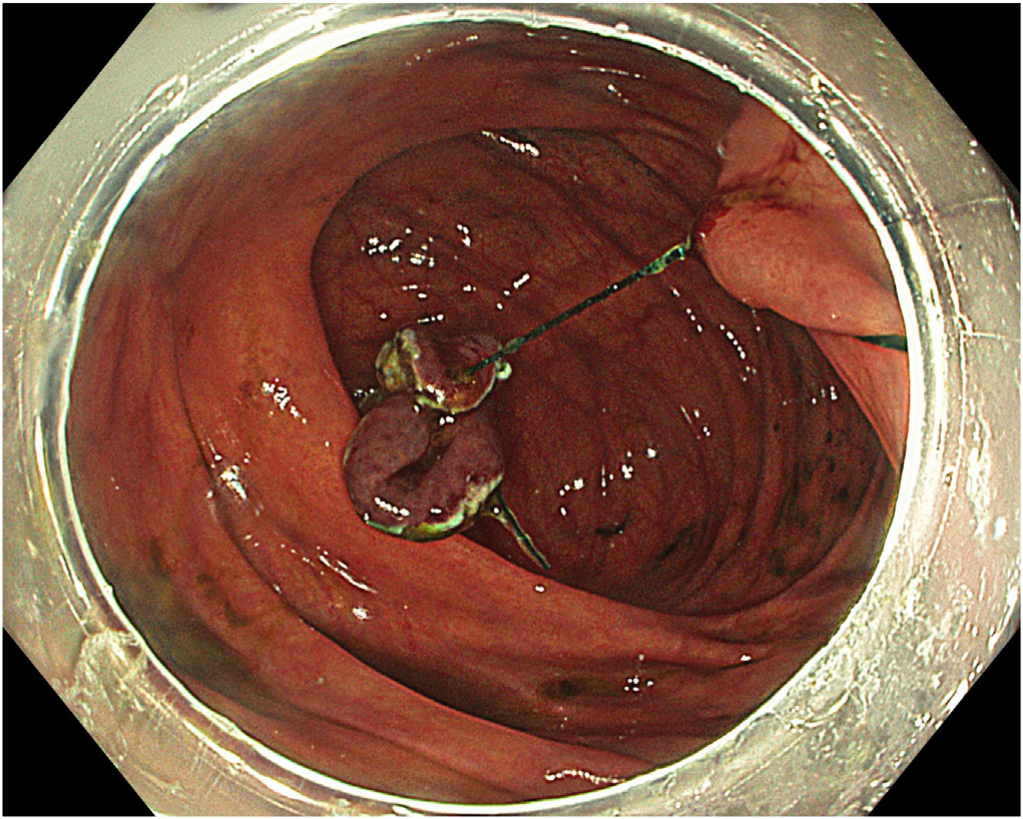
The lesion with a barbed suture applied for traction.

**Figure 5:**
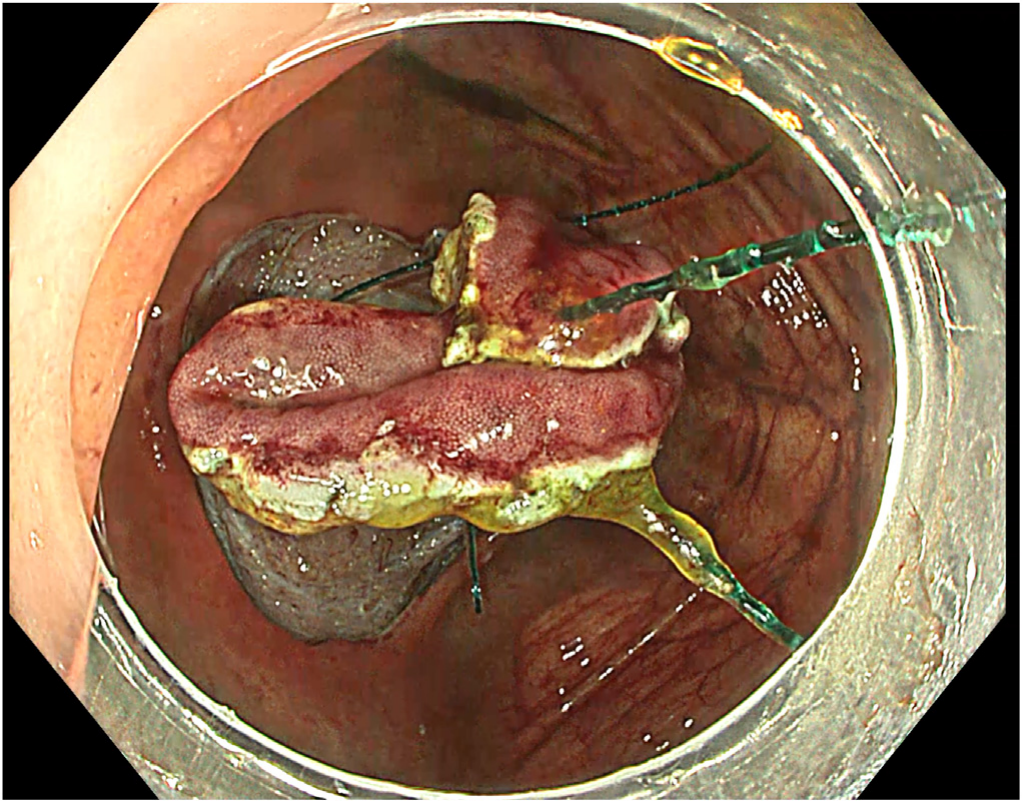
View of the lesion under traction with the barbed suture.

**Figure 6:**
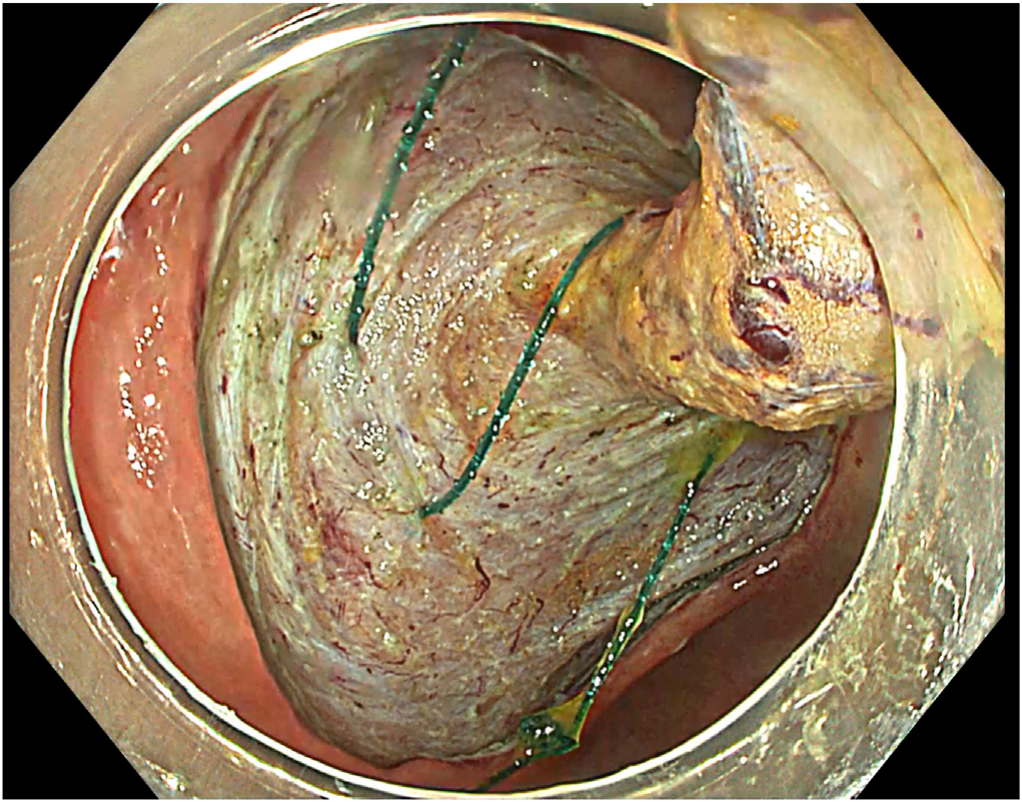
View of the appendix under traction and the application of muscular sutures.

**Figure 7:**
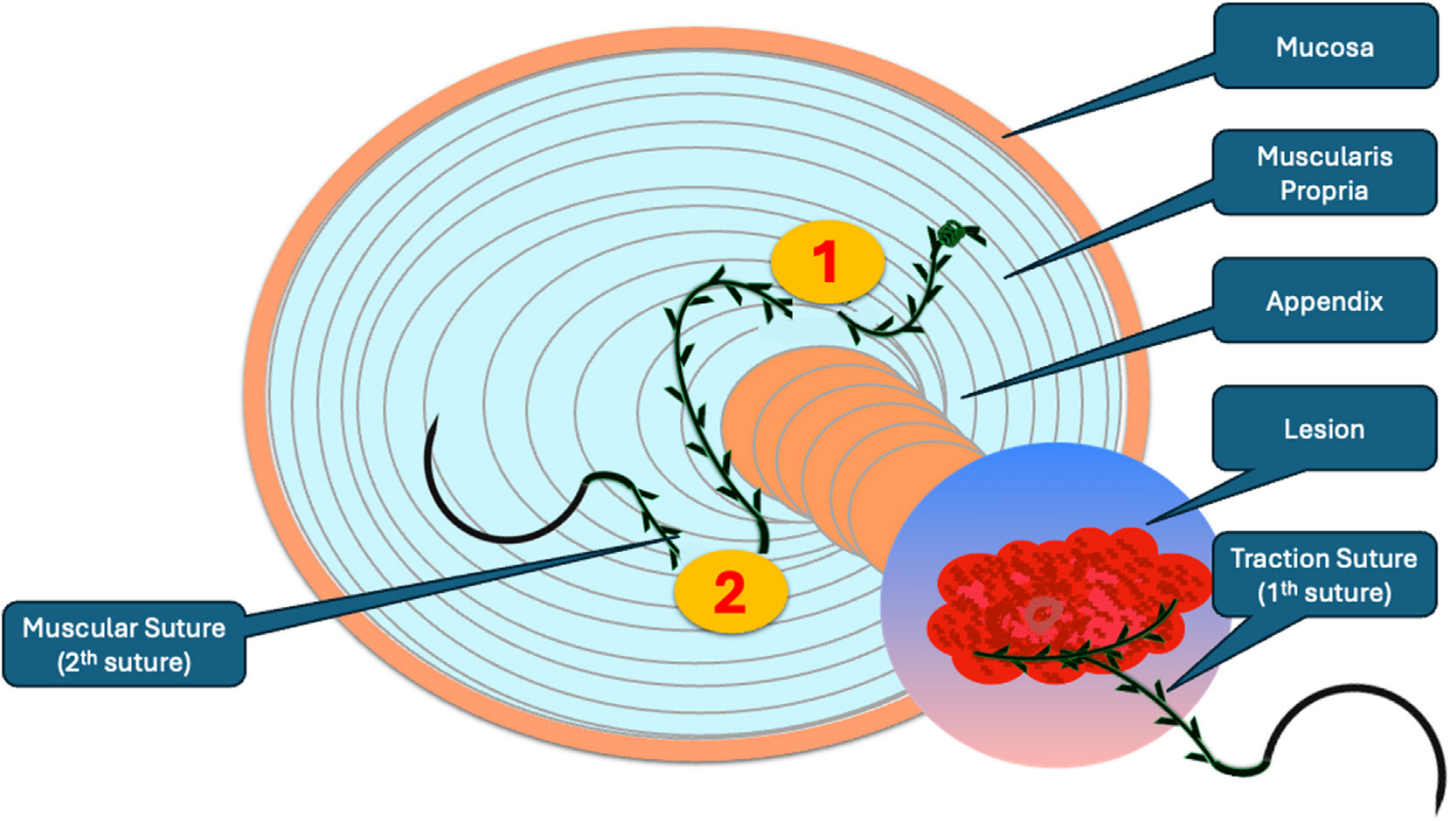
Schematic view of the lesion, appendix, and muscular sutures under traction.

**Figure 8:**
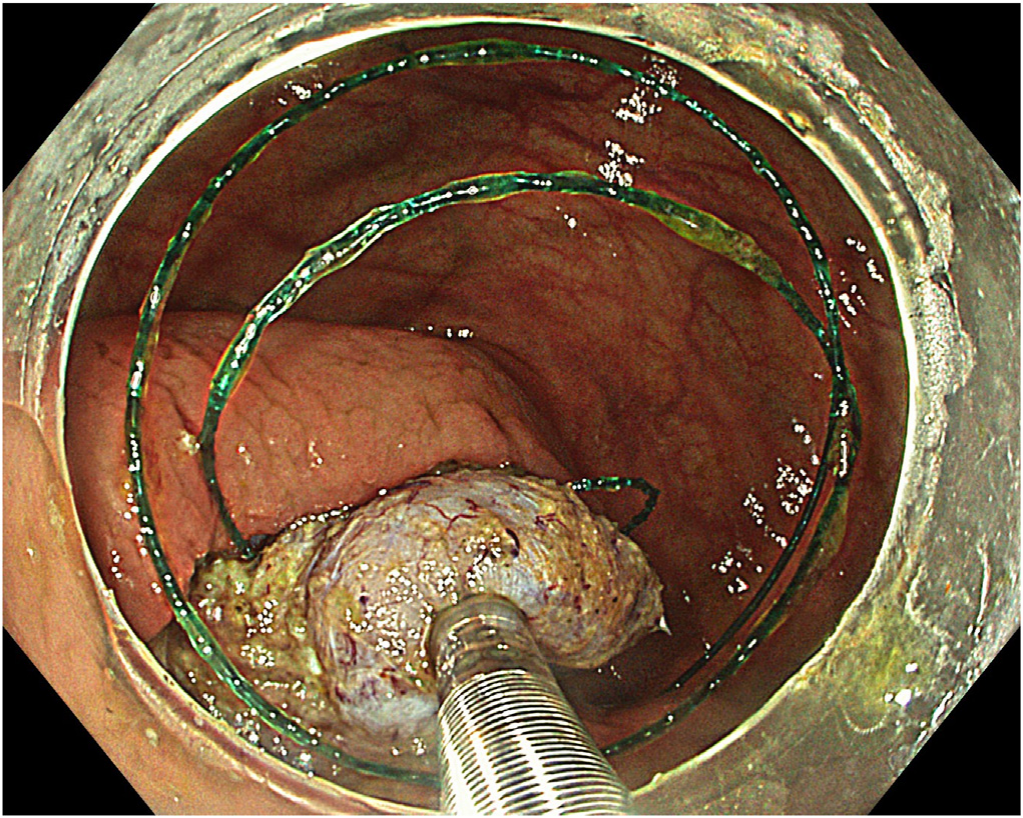
Application of muscular suturing after endoscopic appendectomy.

**Figure 9:**
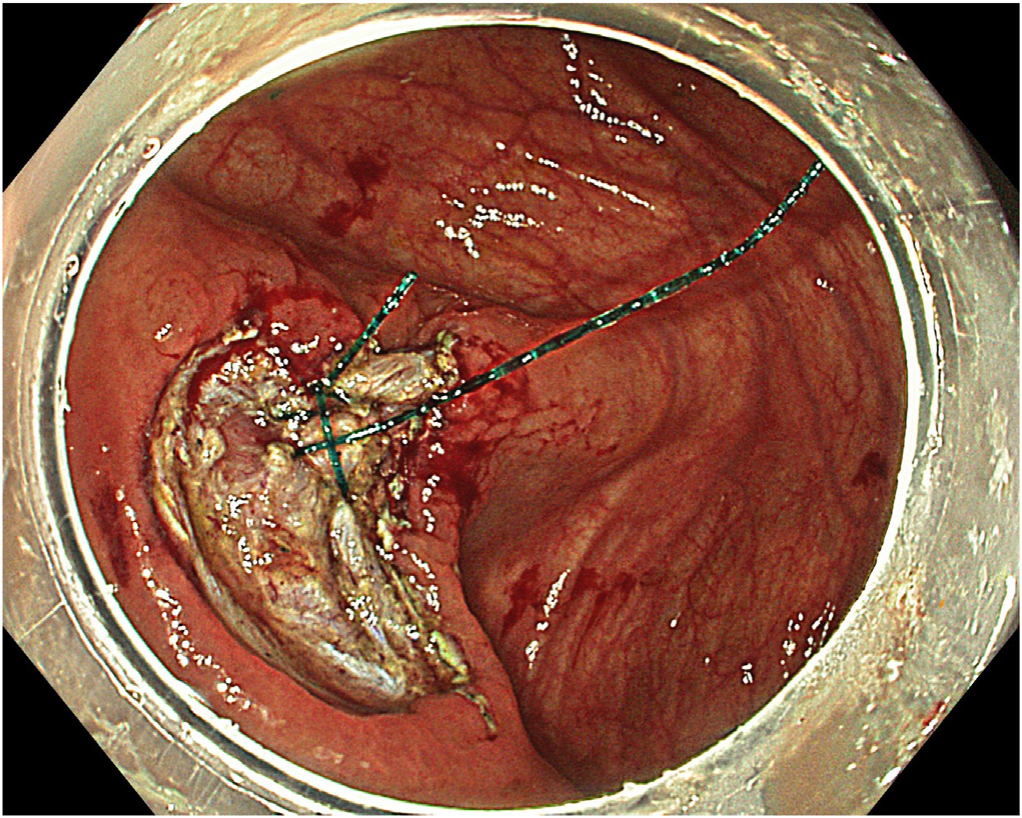
View of the completed muscular suturing.

**Figure 10:**
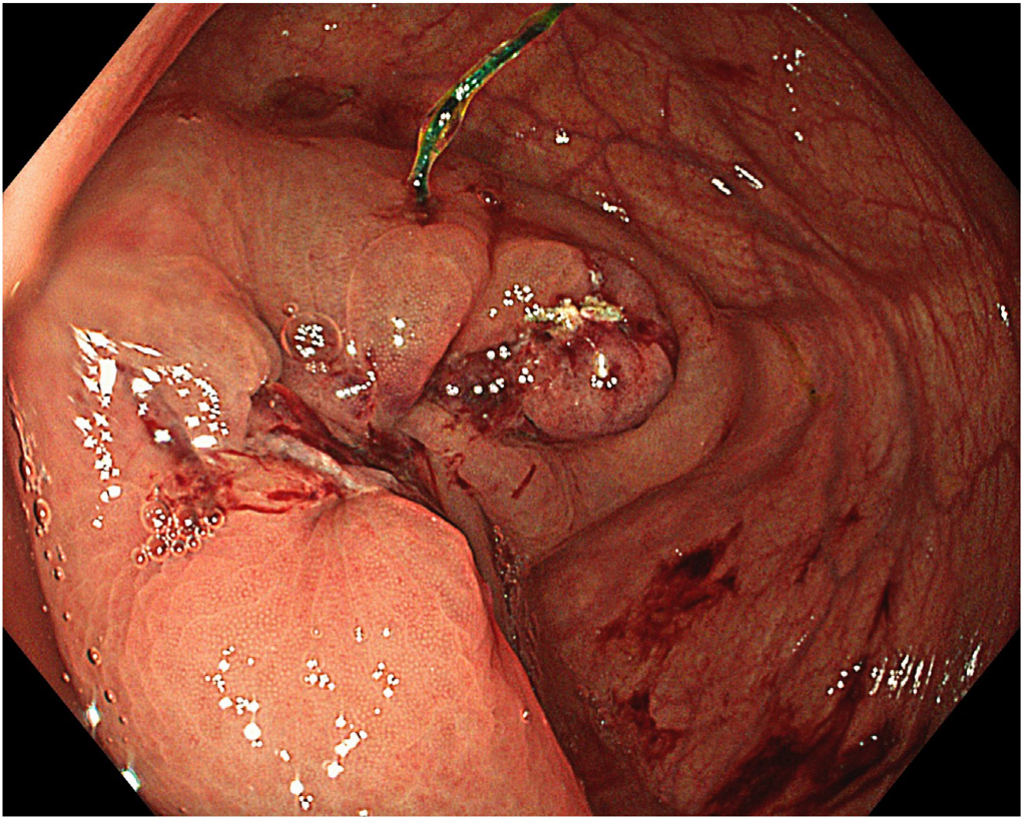
View of the completed mucosal suturing.

**Figure 11:**
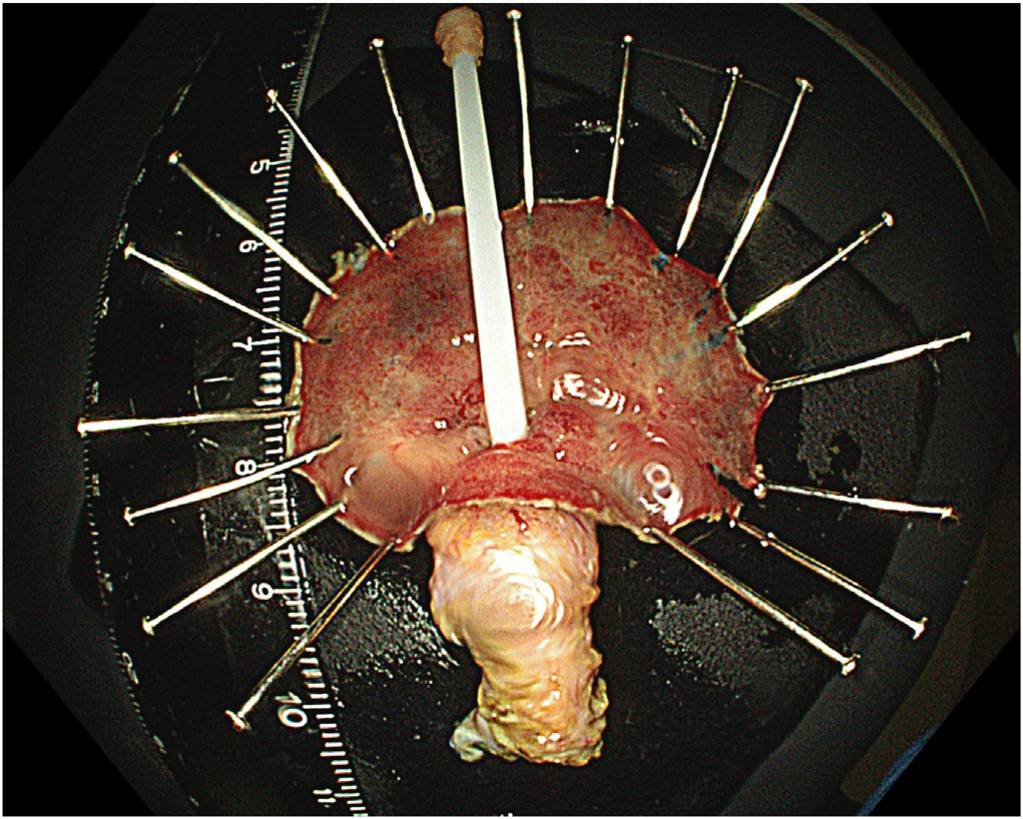
Macroscopic view of the excised cecal laterally spreading tumor and appendix.

**Figure 12:**
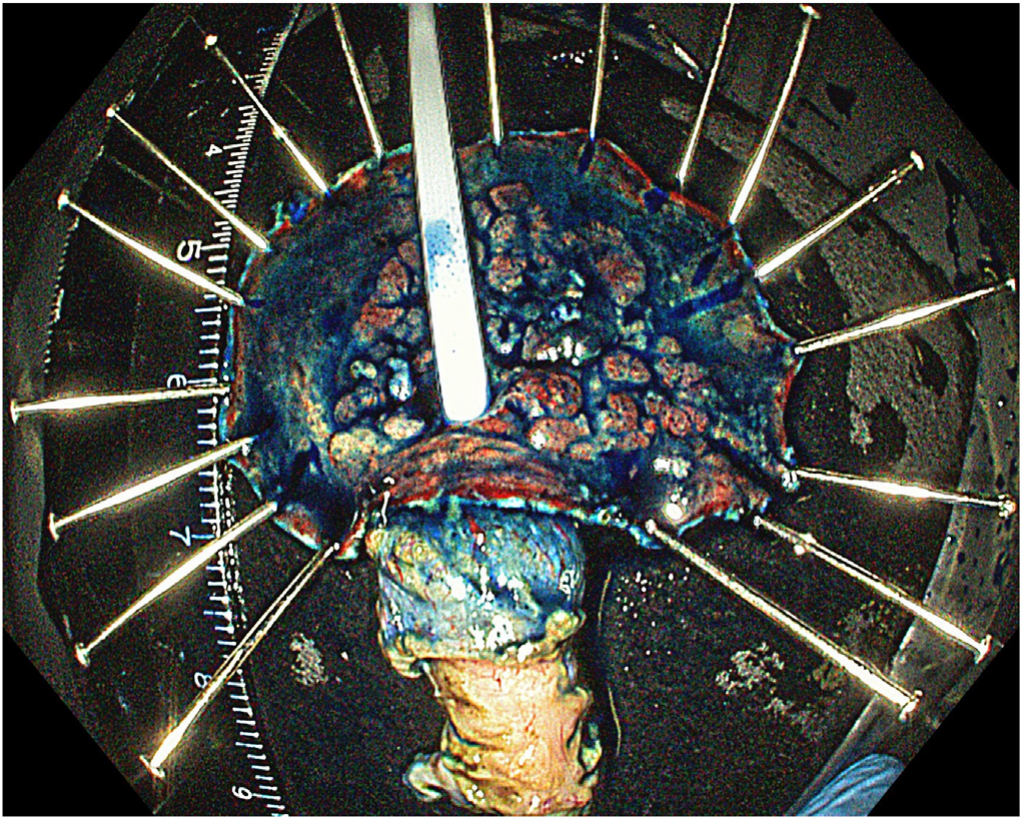
Macroscopic view of the excised cecal laterally spreading tumor and appendix after indigo carmine staining.

**Figure 13:**
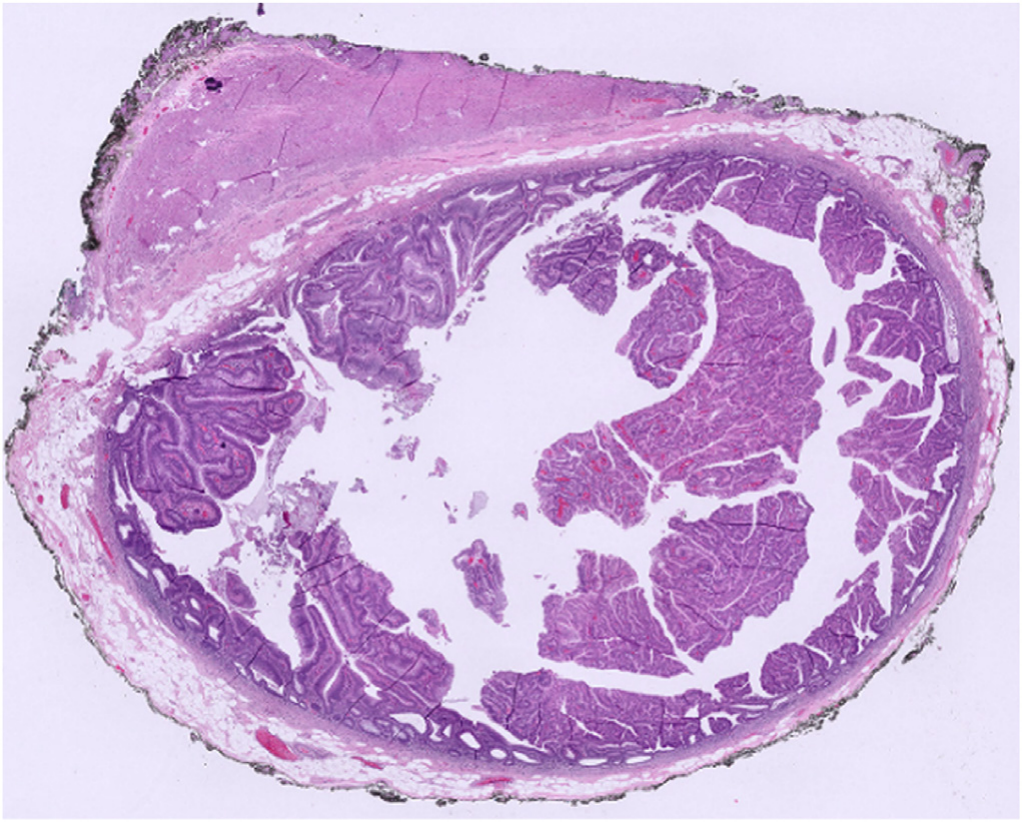
Microscopic view of the lesion. Cross-section of the appendix shows an adenomatous lesion with papillary and villous structures in the mucosa, occupying the entire lumen, whole slide image. (H&E, orig. mag. ×2)

**Figure 14:**
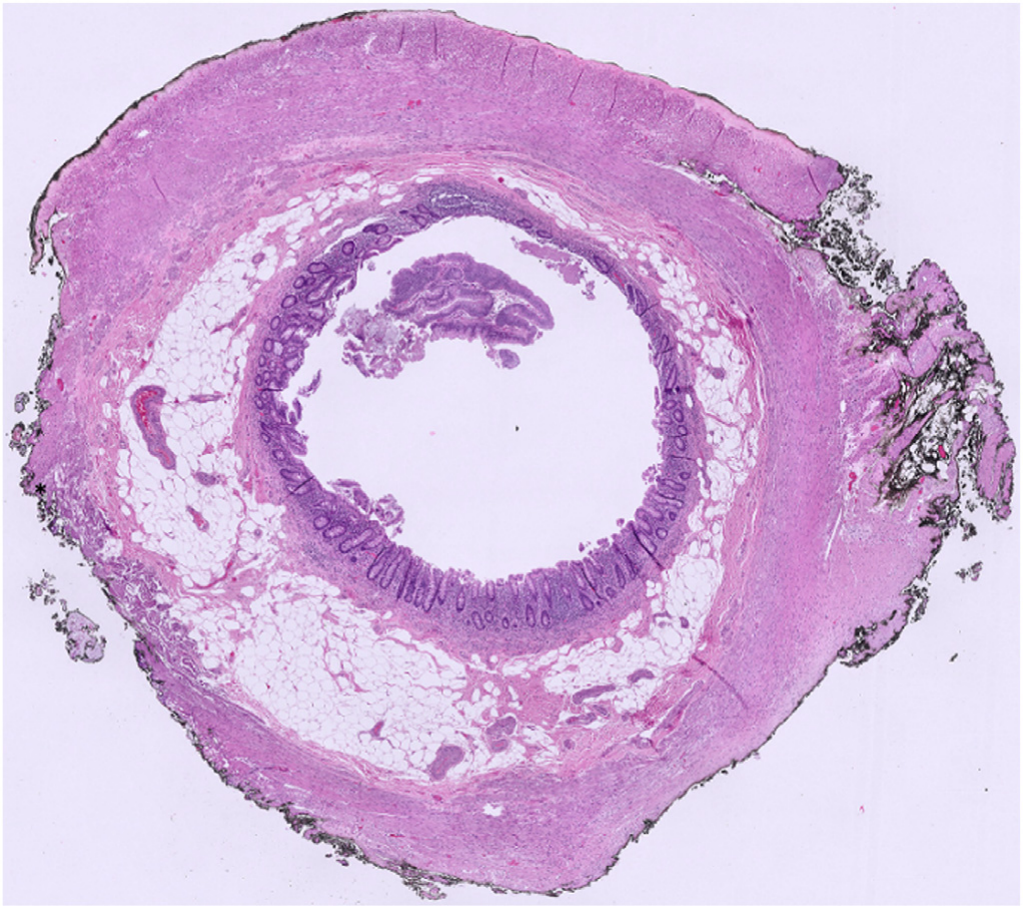
The slice of the appendix representing the interface of normal and adenomatous tissue. A floating strip of lesion is seen in the lumen, whole slide image. (H&E, orig. mag. ×2)

**Figure 15:**
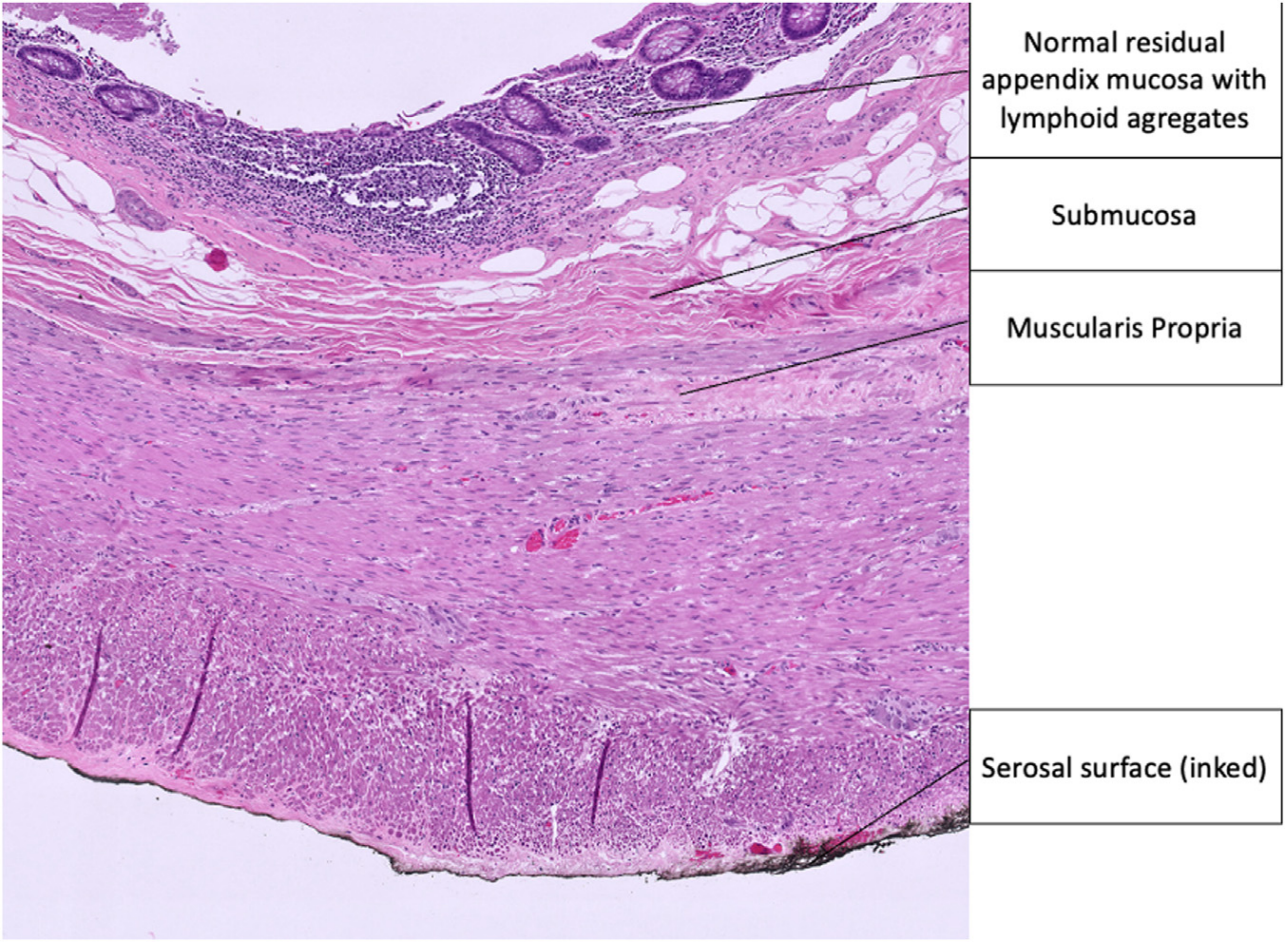
High-power field view of appendiceal wall. (H&E, orig. mag. ×2)

A standard colonoscope fitted with an endoscopic hood (Olympus D-201-11804) was used. A barbed suture (V-Loc 180, absorbable 3-0, CV-23; Medtronic Ltd, Dublin, Ireland) was placed into the hood using a needle holder (Sutuart, FG 260U; Olympus) and advanced to the ascending colon. The suture was anchored to the mucosa in the vicinity of ileocecal valve. Submucosal elevation with a sclerotherapy needle (Needle Master; Olympus) was done. A dual knife (Olympus) was used to make a semicircular mucosal incision on the medial side of the lesion, followed by submucosal dissection until the appendiceal orifice. The same strategy was applied on the lateral side, totally freeing the lesion up to the appendix orifice (Fig. 2). The barbed suture was then inserted through the edges of the released lesion using the needle holder, and traction was maintained by attaching the suture to the proximal fold of the right side of the colon, and a second barbed suture was placed in the exposed muscle tissue around the orifice in resection area to minimize air leakage and provide rapid closure after planned muscular incision (Figure 3, Figure 4, Figure 5, Figure 6, Figure 7). After the first muscular suture, muscular incision was performed at the base of the lesion using the dual knife, and the intra-abdominal cavity was reached. The appendix under traction was carefully dissected from the intra-abdominal space, and the polyp along with the appendix was resected “en bloc.” The previously applied muscular suture was then immediately tightened to close the muscular defect, and continuous suturing was performed to prevent further intra-abdominal contamination and carbon dioxide leakage (Figs. 8 and 9). After muscular closure, the remaining excess suture was cut with endoscopic scissors (Loop Cutter, Olympus). The remaining barbed suture was used to close the mucosal layer (Fig. 10). Thus, both the muscular and mucosal layers were sutured separately (Video 1, available online at www.videogie.org). Total procedure time including ESD, TEA, and muscular and mucosal suturing was 81 minutes. No adverse events occurred during nor after the procedure. The patient was started on a liquid and normal diet on second and third postoperative days, respectively, and discharged by the fourth day. Histopathology confirmed a sessile serrated adenoma extending to appendix with clear margins, and a curative resection was achieved (Figure 11, Figure 12, Figure 13, Figure 14, Figure 15). The appendix length was 3 cm.

## Discussion

Cecectomy, ileocecectomy, or right hemicolectomy are among treatment modalities for premalignant or early-stage malignant lesions of the cecum and appendix. In recent years, with the advent of closure techniques, endoscopy has become a promising alternative.

However, surgical approaches are known to carry a certain risk of morbidity. It has been reported previously that ileocecectomy for appendicitis is associated with adverse events such as abscess, wound-site infection, partial small-bowel obstruction, or pulmonary embolism in 25% of patients; with a need for reoperation in 7; and switching to ileocecectomy upon anastomosis leak in 2 patients.^5^ In addition, cecectomy or right hemicolectomy for benign or malignant lesions of cecum was reported to be associated with postoperative adverse events as high as 23.6%, with fistula being a serious one that lengthened hospital stay and warranted additional operation. In addition, each surgical procedure was noted to take longer than 1 hour.^6^ Among adverse events, port-site herniation was reported to be greater in colorectal surgeries compared with other gastrointestinal ones.^7^ Surgeries for colon cancer carry a 0.4% to 4.2% risk of anastomosis recurrence, with distal resection margin less than 3 cm being the most predictive variable.^8^

Piecemeal EMR may be an alternative for lesions of the cecum and appendix, with a defined risk of recurrence and positive resection margin that necessitate ESD or surgery. In addition, fibrosis secondary to EMR may lower “en bloc” resection in ESD with an increased risk of perforation. Kulaylat et al^9^ reported in their study that 27 of 104 patients referred for EMR needed ultimate curative surgery. Evaluating the boundaries of cecal polyps extending into the appendiceal orifice is anatomically challenging. Achieving R0 resection even with advanced endoscopic methods such as EMR and ESD can be difficult in such cases.^2^ The full-thickness resection device allows for a high rate of R0 resections. However, a 12% to 15% risk of appendicitis after the procedure has been defined.^10^ Recently, endoscopic full-thickness resection and TEA increasingly have been performed. In most cases, various traction techniques, including snare, rubber band, or hemostatic clip, are used to provide rapid and safe resection.^3^ These techniques can be effective; however, they are costly, and snare maneuver within the proximal colon can complicate the procedure.

In our case, a standard barbed suture was used to achieve successful traction without requiring additional endoscopic equipment. We have demonstrated previously that the use of barbed sutures for traction increases dissection speed and contributes to ESD success without adverse event.^4^ Herein, we confirmed that traction of both the lesion and appendix provided faster and safer resection. In this case, once cecal margins were determined via chromoendoscopy, dissection began toward the appendix. Given that the lesion was vague within appendix, the first orifice was isolated circumferentially, then “en bloc” endoscopic appendectomy was completed.

**Figure 16:**
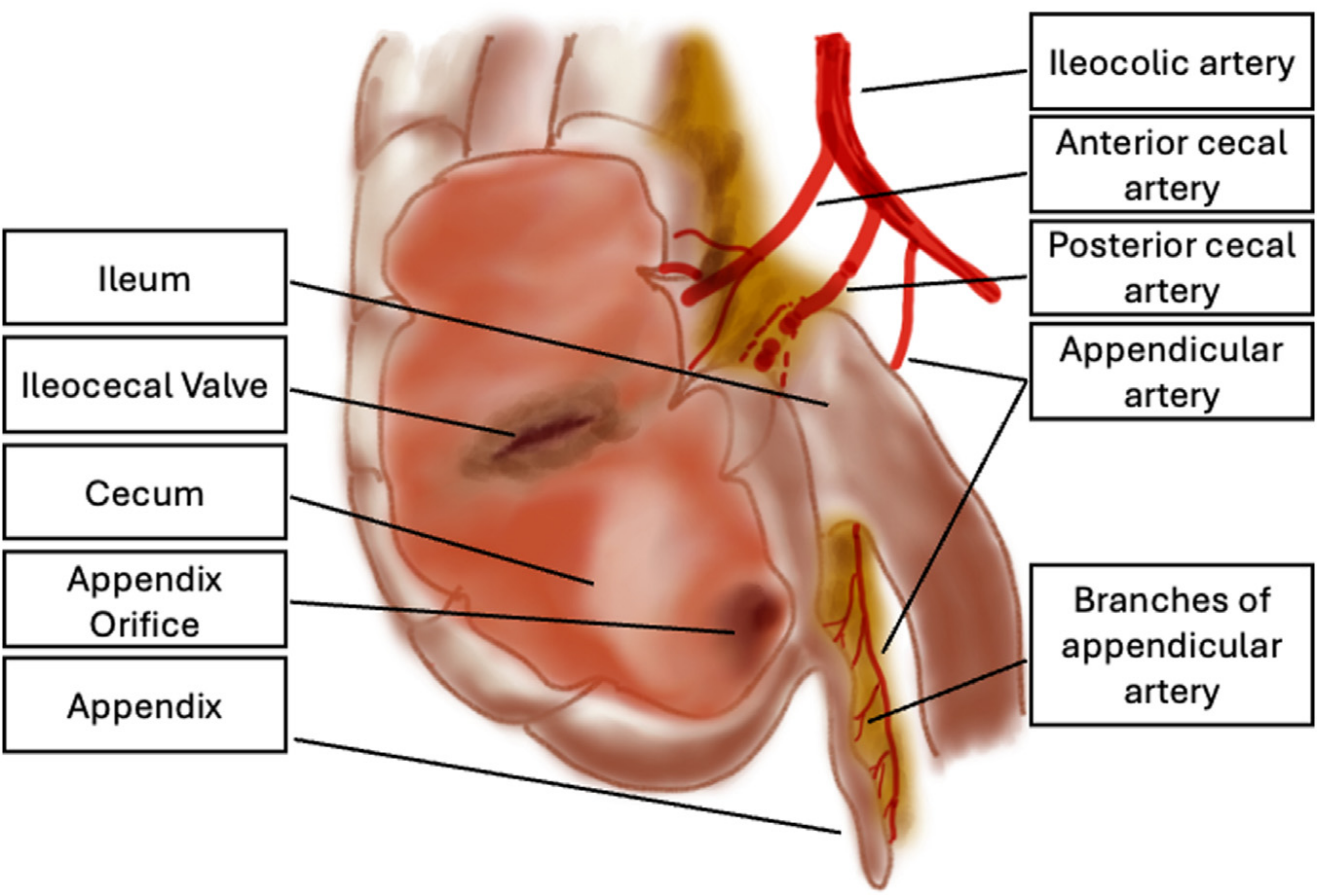
Schematic view of the cecum, appendix, appendicular artery, and its branches.

Major risks during the procedure included damage to appendiceal artery. To minimize this, terminal branches, instead of the main artery, were coagulated and cut with the help of serosal dissection under direct vision. In addition, the traction itself was thought to provide a safer dissection by inverting the appendix (Fig. 16).

The appendix may be located in retrocecal/retrocolic, pelvic, post-ileal, subcecal, pre-ileal, or paracecal positions.^11^ Theoretically, inversion of appendix into the lumen may be challenging, given the serosal network. Although we did not face such an inconvenience in our case, we had considered switching to surgery or laparoscopy and endoscopy cooperative surgery^12^ in case we were not able to invert the appendix under traction despite direct vision.

One of the main challenges during TEA or endoscopic full-thickness resection is the leakage of intraluminal air into the intra-abdominal space. To minimize this, absorbable barbed suture was inserted to the muscular layer in 2 different points and kept ready to close just after muscular dissection. Once appendiceal resection was completed, barbed suture in the muscular layer was pulled, immediately followed by running suturing for complete muscular closure. Therefore, although a 19-gauge venous catheter was held ready, risk of pneumoperitoneum was minimized.

The cecum is well known to be the widest and thinnest part of the colon. Postpolypectomy syndrome, commonly observed after hot-snare polypectomy, EMR, or ESD can easily be confused with delayed perforation.^13^ To minimize the risk of the latter, we applied continuous sutures to both the muscular and mucosal layers after ESD, thereby creating 2 layers of sutures. This approach effectively prevented leakage and reduced the risk of delayed perforation.

In conclusion, in the treatment of laterally spreading tumors involving the appendiceal orifice with unclear border, barbed-suture traction and closure seem to be effective. The adaptation of barbed sutures, frequently used in surgical practice, to endoscopic procedures may provide a minimally invasive alternative in benign and premalignant appendiceal lesions.

## Patient Consent

The patient in this article has given written informed consent to publication of the case details.

## Disclosure

All authors disclosed no financial relationships.

## Supplementary data

MainText-Appendix

Video 1 Transcecal endoscopic appendectomy and endoscopic submucosal dissection with hand-suturing assisted traction and closure technique (8.14 seconds)2
