# Supplementary material for: Transcecal endoscopic appendectomy and endoscopic submucosal dissection with hand-suturing–assisted traction and closure technique
Source: VideoGIE. 2025 Jan 17;10(5):270–6. doi: 10.1016/j.vgie.2025.01.005 (PMC12009091; doi:10.1016/j.vgie.2025.01.005)
Supplement: MainText-Appendix [file mmc1.docx]

**Transcecal Endoscopic Appendectomy and Endoscopic Submucosal Dissection**

**with Hand-Suturing Assisted Traction and Closure Technique**

**Introduction.** The treatment of cecal polyps involving appendiceal orifice and/or its lumen is technically challenging given difficulties in clearly defining the boundaries (1). There is no consensus on the best treatment approach for these polyps. Surgical resection can provide a curative option; however, it carries a certain risk of morbidity. In recent years, several minimally invasive endoscopic treatment techniques have emerged and yielded successful outcomes in the management of appendix polyps. These include traditional polypectomy methods, endoscopic mucosal resection (EMR), endoscopic submucosal dissection (ESD), endoscopic full-thickness resection (EFTR), and transcecal endoscopic appendectomy (TEA)(2, 3). Herein, we present a case of a polyp involving the lumen of the appendix.

**Case.** A 63-year-old male patient underwent colonoscopy (Olympus CF-EZ1500DL, Tokyo, Japan) upon altered bowel habits, and a granular laterally spreading lesion involving cecum and appendix was detected. Lesion border within the appendix was not clearly defined. Lesion surface and vascular pattern were observed to be regular in Texture and Color Enhancement Imaging (TXI), Narrow Band Imaging (NBI), Extended Depth of Field (EDOF) modalities (Figure1). Abdominal tomography revealed neither distant metastasis nor lymph node involvement. Under general anesthesia, the procedure involved hand-suturing traction(4) , ESD, TEA, and closure, respectively. Prophylactic parenteral antibiotic (cefazolin sodium 2g/day and metronidazole 1000 mg/day) was administered and continued until discharge.

A standard colonoscope fitted with an endoscopic hood (Olympus D-201-11804, Tokyo, Japan) was used. A barbed suture (V-Loc 180, absorbable 3-0, CV-23, Medtronic Ltd, Dublin, Ireland) was placed into the hood using a needle holder (Olympus, Sutuart, FG 260U, Tokyo, Japan) and advanced to the ascending colon. The suture was anchored to the mucosa in the vicinity of ileocecal valve. Submucosal elevation with a sclerotherapy needle (Needle Master, Olympus Tokyo, Japan) was done. A dual knife (Olympus, Tokyo, Japan) was used to make a semicircular mucosal incision on the medial side of the lesion, followed by submucosal dissection till the appendiceal orifice. The same strategy was applied on the lateral side, totally freeing the lesion up to the appendix orifice (Figure2). The barbed suture was then inserted through the edges of the released lesion using the needle holder, and traction was maintained by attaching the suture to the proximal fold of the right colon, and a second barbed suture was placed in the exposed muscle tissue around the orifice in resection area to minimize air leakage and provide rapid closure following planned muscular incision(Figure3-7). After the first muscular suture, muscular incision was performed at the base of the lesion using the dual knife, and the intra-abdominal cavity was reached. The appendix under traction was carefully dissected from the intra-abdominal space, and the polyp along with the appendix, was resected “en bloc”. The previously applied muscular suture was then immediately tightened to close the muscular defect, and continuous suturing was performed to prevent further intra-abdominal contamination and carbon dioxide(C0_2_) leakage (Figure8-9). After muscular closure, the remaining excess suture was cut with endoscopic scissors (Loop Cutter, Olympus, Tokyo, Japan). Remaining barbed suture was used to close the mucosal layer (Figure10). Thus, both the muscular and mucosal layers were sutured separately(Video). Total procedure time including ESD, TEA, muscular and mucosal suturing was 81 minutes. No adverse events occurred during nor after the procedure. The patient was started on a liquid and normal diet on second and third postoperative days, respectively; and discharged by the fourth day. Histopathology confirmed a sessile serrated adenoma extending to appendix with clear margins, and a curative resection was achieved (Figure11-15). The appendix length was 3 cm.

**Discussion.** Cecectomy, ileocecectomy or right hemicolectomy are among treatment modalities for premalignant or early-stage malignant lesions of cecum and appendix. In recent years, with the advent of closure techniques, endoscopy has become a promising alternative.

Besides, surgical approaches are known to carry a certain risk of morbidity. It has been previously reported that ileocecectomy for appendicitis complicated with abscess, wound site infection, partial small bowel obstruction or pulmonary embolism in 25% of patients; with a need for re-operation in 7 and switching to ileocecectomy upon anastomosis leak in 2 patients(5). Plus, cecectomy or right hemicolectomy for benign or malignant lesions of cecum was reported to be associated with post-operative adverse events as high as 23,6%, with fistula being the serious one which lengthened hospital stay and warranted additional operation. Also, each surgical procedure was noted to take more than one hour(6). Among adverse events, port site herniation was reported to be higher in colorectal surgeries compared to other gastrointestinal ones(7). Besides, surgeries for colon cancer carry a 0,4-4,2% risk of anastomosis recurrence with the distal resection margin below 3 cm being the most predictive variable(8).

Piecemeal EMR may be an alternative for lesions of cecum and appendix, with a defined risk of recurrence and positive resection margin which necessitate ESD or surgery. Additionally, fibrosis secondary to EMR may lower “en bloc” resection in ESD with an increased risk of perforation. Kulaylat et al, reported in their study that 27 out of 104 patients referred for EMR needed ultimate curative surgery(9). Evaluating the boundaries of cecal polyps extending into the appendiceal orifice is anatomically challenging. Achieving R0 resection even with advanced endoscopic methods such as EMR and ESD can be difficult in such cases(2). The Full-Thickness Resection Device (FTRD) allows for a high rate of R0 resections. However, a 12-15% risk of appendicitis following the procedure has been defined(10). Recently, endoscopic full-thickness resection and TEA have increasingly been performed. In most cases, various traction techniques, including snare, rubber band, or hemoclip, are used to provide rapid and safe resection (3). These techniques can be effective; however, they are costly, and snare maneuver within the proximal colon can complicate the procedure.

In our case, a standard barbed suture was used to achieve successful traction without requiring additional endoscopic equipment. We have previously demonstrated that the use of barbed sutures for traction increases dissection speed and contributes to ESD success without adverse event(4). Herein, we confirmed that traction of both the lesion and appendix provided faster and safer resection. In this case, once cecal margins were determined via chromoendoscopy, dissection began towards appendix. Given the lesion was vague within appendix, first orifice was isolated circumferentially, then “en bloc” endoscopic appendectomy was completed.

Major risk during the procedure included damage to appendiceal artery. To minimize this, terminal branches, instead of the main artery, was coagulated and cut with the help of serosal dissection under direct vision. Besides, the traction itself was thought to provide a safer dissection by inverting the appendix (Figure 16).

Appendix may be located retrocecal/retrocolic, pelvic, post-ileal, subcecal, pre-ileal or paracecal positions(11). Theoretically, inversion of appendix into the lumen may be challenging given serosal network. Although we did not face such an inconvenience in our case, we had considered switching to surgery or laparoscopy and endoscopy cooperative surgery(12) in case we could not be able to invert appendix under traction despite direct vision.

One of the main challenges during TEA or EFTR is the leakage of intraluminal air into the intra-abdominal space. To minimize this, absorbable barbed suture was inserted to muscular layer in two different points and kept ready to close just after muscular dissection. Once appendiceal resection was completed, barbed suture in the muscular layer was pulled immediately followed by running suturing for complete muscular closure. So, although a 19G venous catheter was held ready, risk of pneumoperitoneum was minimized.

The cecum is well-known to be the widest and thinnest part of the colon. Post-polypectomy syndrome, commonly observed after hot-snare polypectomy, EMR, or ESD can easily be confused with delayed perforation(13). To minimize the risk of the latter, we applied continuous sutures to both the muscular and mucosal layers after ESD, thereby creating two layers of sutures. This approach effectively prevented leakage and reduced the risk of delayed perforation.

As a conclusion, in the treatment of laterally spreading tumors involving the appendiceal orifice with unclear border, barbed-suture traction and closure seem to be effective. The adaptation of barbed sutures, frequently used in surgical practice, to endoscopic procedures may provide a minimally invasive alternative in benign and premalignant appendiceal lesions.

**REFERENCES**

1. Amini A, Koury E, Vaezi Z, Talebian A, Chahla E. "Obscure" Appendiceal Orifice Polyps Can Be Challenging to Identify by Colonoscopy. Case Rep Gastroenterol. 2020;14(1):15-26.

2. Vargas JI, Teshima CW, Mosko JD. Management of Periappendiceal Orifice Polyps. Clinical Gastroenterology and Hepatology. 2020;18(11):2425-9.

3. Keihanian T, Khalaf MA, Aloor FZ, Zamil DH, Jawaid S, Othman MO. Transcecal endoscopic appendectomy for management of complex appendiceal polyps extending into the appendiceal orifice. Endosc Int Open. 2024;12(8):E932-e9.

4. Aslan F, Ozer S, Demirdogen V. Hand-Suturing Assisted Traction Technique and Closure in Colon Endoscopic Submucosal Dissection. VideoGIE. 2024. https://doi.org/10.1016/j.vgie.2024.10.010

5. Lane JS, Schmit PJ, Chandler CF, Bennion RS, Thompson JE, Jr. Ileocecectomy is definitive treatment for advanced appendicitis. Am Surg. 2001;67(12):1117-22.

6. Abdalla S, Meillat H, Fillol C, Zuber K, Manceau G, Dubray V, et al. Ileocecal Valve Sparing Resection for the Treatment of Benign Cecal Polyps Unsuitable for Polypectomy. Jsls. 2021;25(2).

7. Owens M, Barry M, Janjua AZ, Winter DC. A systematic review of laparoscopic port site hernias in gastrointestinal surgery. Surgeon. 2011;9(4):218-24.

8. Huang F, Jiang S, Wei R, Xiao T, Wei F, Zheng Z, et al. Association of resection margin distance with anastomotic recurrence in stage I-III colon cancer: data from the National Colorectal Cancer Cohort (NCRCC) study in China. Int J Colorectal Dis. 2024;39(1):105.

9. Kulaylat AS, Boltz MM, Moyer M, Mathew A, McKenna K, Messaris E. Management of Large Cecal Polyps: When Can the Ileocecal Valve Be Spared? Dis Colon Rectum. 2018;61(9):1089-95.

10. Obri M, Ichkhanian Y, Brown P, Almajed MR, Nimri F, Taha A, et al. Full-thickness resection device for management of lesions involving the appendiceal orifice: Systematic review and meta-analysis. Endosc Int Open. 2023;11(9):E899-e907.

11. Constantin M, Petrescu L, Mătanie C, Vrancianu CO, Niculescu AG, Andronic O, et al. The Vermiform Appendix and Its Pathologies. Cancers (Basel). 2023;15(15).

12. Suzuki K, Saito S, Fukunaga Y. Current Status and Prospects of Endoscopic Resection Technique for Colorectal Tumors. J Anus Rectum Colon. 2021;5(2):121-8.

13. Hirasawa K, Sato C, Makazu M, Kaneko H, Kobayashi R, Kokawa A, et al. Coagulation syndrome: Delayed perforation after colorectal endoscopic treatments. World J Gastrointest Endosc. 2015;7(12):1055-61.
